# Mobile Phone Call Data as a Regional Socio-Economic Proxy Indicator

**DOI:** 10.1371/journal.pone.0124160

**Published:** 2015-04-21

**Authors:** Sanja Šćepanović, Igor Mishkovski, Pan Hui, Jukka K. Nurminen, Antti Ylä-Jääski

**Affiliations:** 1 Department of Computer Science, Aalto University, Helsinki, Finland; 2 Faculty of Computer Science and Engineering, University Ss. Cyril and Methodius, Skopje, Macedonia; 3 Department of Computer Science and Engineering, Hong Kong University of Science and Technology, Hong Kong, China; University of Namur, BELGIUM

## Abstract

The advent of publishing anonymized call detail records opens the door for temporal and spatial human dynamics studies. Such studies, besides being useful for creating universal models for mobility patterns, could be also used for creating new socio-economic proxy indicators that will not rely only on the local or state institutions. In this paper, from the frequency of calls at different times of the day, in different small regional units (sub-prefectures) in Côte d'Ivoire, we infer users' home and work sub-prefectures. This division of users enables us to analyze different mobility and calling patterns for the different regions. We then compare how those patterns correlate to the data from other sources, such as: news for particular events in the given period, census data, economic activity, poverty index, power plants and energy grid data. Our results show high correlation in many of the cases revealing the diversity of socio-economic insights that can be inferred using only mobile phone call data. The methods and the results may be particularly relevant to policy-makers engaged in poverty reduction initiatives as they can provide an affordable tool in the context of resource-constrained developing economies, such as Côte d'Ivoire's.

## Introduction

Human dynamics is a research branch of complex systems that was particularly influenced by the availability of (big) data that carry location information: from the traveling bank-notes [1], mobile phone logs [2–4] or traveling smart cards [5], to the on-line check-ins with geolocations obtained from social networks, such as Foursquare [6] and Twitter [7]. The location-based datasets have also proven useful for practical studies in several important sectors; for example, for modeling and future planing, and real-time and aftermath analysis in disaster response and disaster risk reduction, and for health, socio-economic and transportation sector. A potential is also recognized for a planet-scale mobility measurement [8] through opening to researchers and combining the different big datasets carrying location information.

Mobile phone datasets have shown to be particularly useful and popular as they provide temporal and spatial information on a scale and granularity that was not available to researchers before. The call detail record (CDR) datasets enabled researchers in the field of *theoretical human dynamics* to discover power-law distributions to characterize many of the human activities that small datasets could not reveal. The seminal paper by Barabási in 2005 gave an explanation of the bursty nature for many of the human activities [9], while in later works, he and his colleagues discuss predictability of human behavior [10], and propose a universal model for mobility and migration patterns [11]. The radiation model for mobility and migration patterns presented in [11] is based on the observations from the U.S. census data on commuting as well as on mobile communication data. The authors explain that the new model avoids many limitations and provides many improvements compared to the gravity model [12, 13] that was used earlier in a variety of fields that require predicting population movement. Using the same CDR dataset for Côte d’Ivoire [14] that we use in this work, the researchers recently showed improved prediction of population movement devising a new, communication model [15], based solely on CDR data.

When it comes to *practically-oriented human dynamics* research, the authors in [16] show the potential for urban studies and planning by using location-based services, in particular from mobile phone datasets from the city of Milano. Authors in [17] provided partial solution to the traffic congestion in the city of Abidjan using the CDR dataset for Côte d’Ivoire [14]. Their analysis shows that by adding four additional routes and extending one existing route the people in Abidjan will reduce their travel time by 10%. Another field that has great benefit from time-sensitive information obtained from CDRs is the disaster response and disaster risk reduction, in terms of fast resource allocation and directing the emergency aid, as well as in analysing the people’s movement and migration after some emergency situation [18]. The results in [19] emphasized that social bounds are crucial for people’s movement in case of earthquake. This kind of analysis could be further used to estimate the post-catastrophic population situation in a given region and to plan emergency aid more efficiently. In [2] the authors show how emergency situations can be detected by only observing normal collective calling patterns and alerting those patterns that exceed threshold around mean activity.

Today, especially in developing countries, the process of obtaining relevant real-world indicators is a hard task that needs a lot of expertise and resources. Thus, it is desirable if the results from CDR analysis can be used as proxy indicators to estimate and give insights of the socio-economic situation of one country. For instance, it has been shown that the diversity of individuals’ relationships can be used as an indicator of the economic development of certain communities. The more the diverse they are the better the access exist to social and economic opportunities [20]. Another research in Latin America showed that the reciprocity of communications, the physical distance with the contacts and the area in which people move is tightly connected to the socio-economic level of a person and the expenses [21]. In [22] using the CDR dataset for Côte d’Ivoire [14], authors have detected and validated the poverty levels of the 11 different regions. Thus, they again prove that the provided CDR dataset can be used as a proxy indicator for assessing the health, education, living standard and the threat from violence.

In this work, using mobility and calling traces from the CDR dataset for Côte d’Ivoire [14], we analyze different temporal and spatial mobility patterns that occur in the different regions in Côte d’Ivoire. Then we present diverse insights that are revealed about the country from obtained patterns. The time-variance of the mobility pattern can be used to detect *important events* that happened in the observed period. Moreover, when mobility is compared with the number of calls people have made, we have obtained positive correlation, whereas this was not the case with the duration of calls on an average day. The spatial-variance of calling frequency identifies rural and regions lacking in electricity, as well as the division between the west and east parts of the country, captured by night-life activity. The spatial dimensions of the mobility showed high correlation between the *census data* and the extracted number of people whose home is in one of the 11 regions. Moreover, we succeed in proxying the census on a much finer scale, i.e. on a sub-prefecture level (there are 255 sub-prefectures in Côte d’Ivoire). The movement graph and the spatial-variance of the radius of gyration identify the *economically important regions* of Côte d’Ivoire, as well as the regions that are lacking with service infrastructure. The negative correlation between the number of workers in one region, which commute from other regions and the Multidimensional Poverty Index (MPI), shows that the provided data and the methods can be a reasonably good poverty proxy indicator. Besides the economy, we show that the *administrative division* of one country has a great impact on the commuting behavior of the citizens. Using the commuting *O*−*D* (origin-destination) matrix we have presented the economic ties between the administrative regions and moreover, we have classified the regions according to their employment importance. From the results on the commuting distance (i.e. the distance that the workers have to travel each day) on a regional level, and knowing the road infrastructure and the means of transport in Côte d’Ivoire, government and other stakeholders could obtain the carbon footprint of the country or plan future traffic optimizations and gas station positioning.

The work in this paper proves that the spatio-temporal data extracted from mobile phone activity could be used as a proxy indicator for different socio-economic segments and field surveys. Considering that we use only a simple CDR dataset from a developing country, the diversity of insights that can be generated about the country is impressive. Furthermore on a practical side, the provided real-time proxy indicators can guide future policies and help boost economic development on a finer spatial scale.

## Materials and Methods

We analyze mobile phone dataset provided by the Orange operator, Côte d’Ivoire, for Data for Development Challenge (D4D) [14]. The finest division of Côte d’Ivoire we work on is on a sub-prefecture level. The state is divided in 255 sub-prefectures which are administrative units. The used dataset contains the geographic centers of these sub-prefectures. Based on the regional economy level the whole country is divided in 10 development poles [23]. Because Abidjan is quite economically superior to other cities and regions in Côte d’Ivoire we have extracted it from the South pole and counted it as a separate pole. Thus, we work with the country division on 11 development poles, that we call regions throughout the text.

The datasets shared as part of the D4D challenge are preprocessed by Orange (details of preprocessing are given in [14]) from an original dataset containing communication of 5 million Orange users for a 5 months period, covering a significant portion of the total 20 million population, and a long enough period to observe spatial and temporal patterns in calling activity and dynamics. In detail, Orange shared 4 different datasets preprocessed from the original dataset, and we focus on the following two:

1. **Antenna communication dataset:** geolocation coordinates for 1231 antennas that belong to the Orange operator in the country are provided together with their mutual hourly communication (comprising the number of calls and their total duration between the pairs of antennas). We model this dataset as a network of communication with antennas as nodes. The communication is represented by the weight of the edges equaling the number of calls between two antennas, or, in another case, the total duration of the calls. By aggregating the data from these kind of networks, we obtained hourly footprint for calling frequency and duration for the whole country.
2. **Low resolution user trajectories dataset:** during the whole 5 months for half a million users we have the time and the sub-prefecture in which the the call is placed. This dataset enables us to define the home sub-prefecture for a user and thus divide the user base into groups by sub-prefectures. Our approach for inferring home and work locations is explained in the section below. It is stochastic and simple, but validation based on census population density data indicates high accuracy of the approach. After such division, we calculate different types of statistics for users from different sub-prefectures and compare these statistics. We also tackle this dataset from another point: by extracting and aggregating commuting patterns between different sub-prefectures. In this way we can form a directed network with sub-prefectures as nodes having the links between them with a weight based on the number of found commuting patterns. We apply a community detection algorithm [24] on this network to discover the commuting regions in the country for the 5 months period. Analysis of differences in the timing of the calls for the different regions is also presented.

### Inferring home and work locations

**Fig 1:**
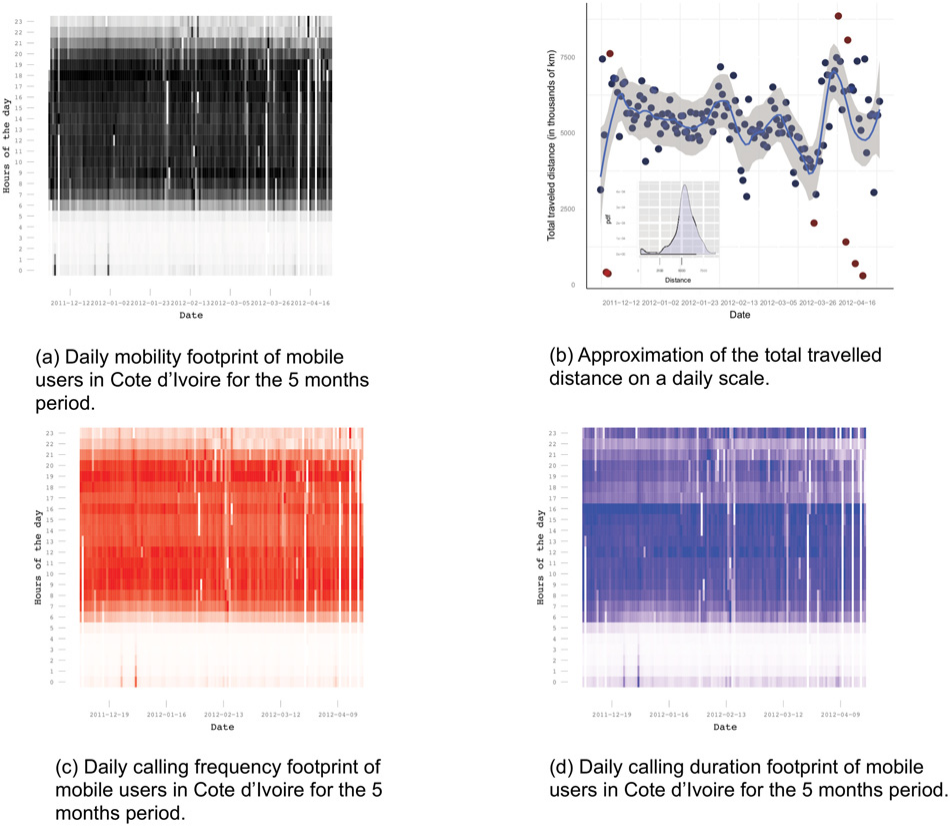
Mobility vs. calling frequency and duration. (a) Daily mobility footprint. (b) Total daily traveled distance. (c) Daily calling frequency footprint. (d) Daily calling duration footprint.

Our aim is to obtain the proxy for the census data on a sub-prefecture level. Similarly to [25, 26], we calculate important locations (home and work) based on the frequency of calls users make in the different locations during predefined home or work hours window. For each user, we rank sub-prefectures based on the number of calls the user makes in them in the period of the day when it is expected to find him at home: during the non-working hours on weekdays (19h to 05h), and during the whole days on the weekends. The sub-prefecture with the most recorded calls is assigned to be the home for a given user. We decide to include weekends in our home calculation using following reasoning: even though people might take some weekends for traveling, our dataset spans long enough period to observe for more of the weekends that the people spend at home than they would travel. Our inherent assumption (and a possible limitation of our approach) is that, in general, more of the weekends are spent not traveling, for majority of the users. A similar approach is applied for finding user’s workplace sub-prefecture, only using the weekday periods between 05h and 19h (we found that this is the widest daily interval capturing commuting, see Fig 1(a)).

In order to account only for the users for whom we have enough fine-grained mobility traces to infer home and work location, we applied two type of filters. The first filter threshold we applied is that a user made at least 30 calls during the whole period for each of the important locations (home and work). The second filter boundary is that the most frequent location, found for either home or work, accounts for at least 50% of the total calls during the home or work hours, respectively. With such a filtering, we are left with the dataset comprising 80% of the original users. Despite some criticism being raised for using CDR data for analysing some aspects of human mobility, it has been confirmed that the calling activity creates a suitable sample for inferring home and work locations [27].

### User mobility statistics

For characterizing mobility of different regions, we calculate diverse statistics for each user and aggregate the values for the users who have the same home locations.

The *daily trajectory* for each user is obtained based on the locations of consecutive calls the user has made during the day. In this way, we might not capture the full path that users make, but we do track the change of location whenever they make calls afterwards. Due to this limitation and since the mobility on a sub-prefecture level is coarse-grained, our focus is on characterizing aggregate user behavior and differences, rather than single user’s mobility. The average length is calculated for the user’s daily trajectories for the 5 months.

As for calculating the *radius of gyration*, we apply the approach presented in [28].

For all the users living in a sub-prefecture of interest, we also observe their *movement graph* comprising of all the daily trajectories in the period. Frequent sub-graph mining tool gSpan [29] is then applied on the set of user movement graphs to find frequent user routes in each region.

We define *commuting trip* to be a trip taken during one day starting from a certain sub-prefecture, going through any set of sub-prefectures and coming back to the starting sub-prefecture by the end of the day. Additionally, such a trip must be taken at least 3 times during a week by the user, in order to be counted.

## Results and Discussion

### Mobility relationship to calling frequency and duration

In Introduction we discussed the existing analyses showing how mobility patterns from mobile call traces can be used as a proxy indicator for traffic conditions according to which transport modeling can be done. Moreover, besides regular mobility patterns, the analyses can point out some extreme events in a given period causing reduced traffic or excessive traffic that shifts the pattern. For instance, Fig 1(a) shows that only small portion of Côte d’Ivoire population goes to work at 05h, after which more and more people start to go to work. The morning rush hour in the country is between 08h and 09h. Then, the nation’s mobility reduces between 09h and 12h, and has quite moderate dynamics between 13h and 15h. The second, afternoon rush hour happens around 18h. In Fig 1(b) we show the total traveled distance by all users, in thousands of km per day. The total users’ traveled distance per day follows a normal distribution (see the inner-plot of Fig 1(b)) with a mean value of 5.3 millions of km. The red dots in the outer-plot in Fig 1(b), show some significant anomalies with respect to the mobility. The lower mobility, could be due to some problems within the mobile operator network or with the data provided in that day, road blocks, riots, etc. However, the high extreme anomalies represent some important events that happened in Côte d’Ivoire on that day or period. For instance, the visual representation in Fig 1(a) underlines three different significant time periods in Côte d’Ivoire: i) on 11-th of December 2011 the parliamentary elections in Côte d’Ivoire were held; ii) at the end of January and beginning of February, the Africa Cup of Nations was held; and iii) the most turbulent period is in the beginning of April, when we have the following events: military victory of the UN-backed pro-Ouattara forces in April 2012; the Buoake Carnival in the end of March and the first week of April; the public festival Fete du Dipri in April 2012; the military coup in Mali and ECOWAS summit in Abidjan. Additionally, Fig 1 displays the importance of global annual events in Côte d’Ivoire, such as New Year’s Eve, Christmas (25th December) and Easter (8th April), on the dates for which, we can clearly see the increased activity in the hours after midnight.

Concerning the weekdays, the mobility is largest on Fridays, with around 35% increase from the most leisure day, i.e. Sunday. We think that the explanation is that on Fridays people tend to travel more for weekend vacancies or visiting their relatives.

Similar pattern was obtained when analyzing the daily mobility footprint of the mobile users. The results from Fig 1(a) and 1(c) show intuitively expected correlation (*c* = 0.84 and *p* < < 0.01). These promising results presented on a coarse-grain level when combined with the analysis presented in [2, 15] should be used as an incentive for deeper microscopic mobility analysis that will aim to answer the question: Is mobility promoting social activity and communication?

In Fig 1(d) we see the calling duration pattern for the whole period divided per hours of the day. This pattern shows different user behavior from the results for mobility and the calling frequency. The result clearly shows that people tend to talk longer in the period between 12h and 16h, which could be due to the fact that in this period most of the business related conversations occur, and business conversations tend to last longer. This result differs from the one in [30], where the authors show linear correlation between the number of calls and the duration of calls for each antenna. In addition, we see a small peak at 23h, that leads to a conclusion that maybe the operator offers some special tariffs after 23h, or that people want to have longer relaxing talks with the closest ones in the evening hours.

**Fig 2:**
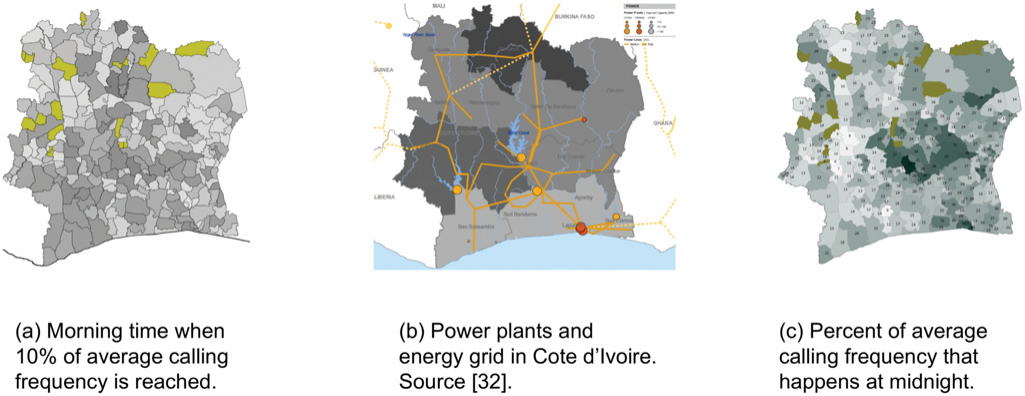
Call timings statistic. (a) Morning 10% calling frequency reaching hour. (b) Energy system in Côte d’Ivoire. (c) Midnight calling frequency.

In the following, we have analysed the diversity of the timings of calls during the day and have represented the results in Fig 2. Fig 2(a) uses a grayscale map to represent how the rural and northern areas reach morning calling frequency increase to the 10% of the average calling frequency at an earlier time (lighter color) compared to the cities and the south (darker color). One explanation is that the people in those areas live more according to the sunrise and “time as measured by a clock has little relevance” as people wake up early in rural areas in developing countries with hot climate “to do their most arduous tasks before it gets too hot” [31]. Additional reason in the case of Côte d’Ivoire is that those regions lack electrical energy. See Fig 2(b) showing that the north suffers from power generation, as well as, power distribution.

Fig 2(c) captures night-life pattern, representing percent of its average calling frequency that the sub-prefecture has at midnight hour. As expected, the cities have the larger percent. Perhaps less expected, our analysis also indicates that the eastern part of the country tends to have more activity at night hours compared to the western. As in [32] and [33], the results show clear division between the east and the west part of the country, mainly due to economical interests, cultural bounds, country infrastructure and the ethnic divide.

### Mobile communication as a fine-grained proxy census indicator

**Fig 3:**
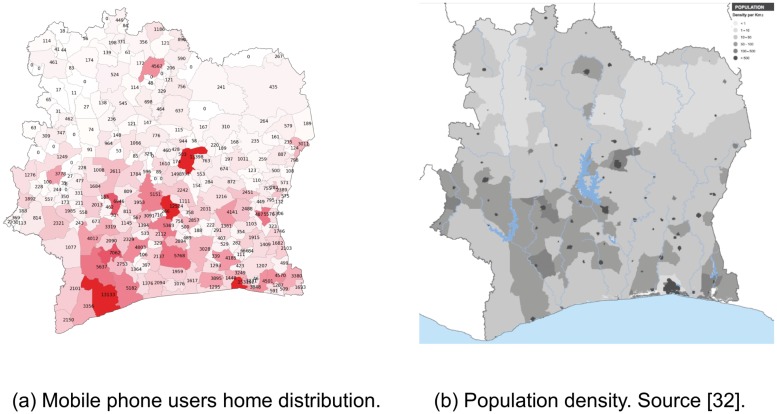
Population density vs. user home distribution. (a) Users home distribution. (b) Population density.

The distribution of the number of found users per sub-prefecture, see Materials and Methods, is not even (Fig 3), a result we would expect, since the population density and mobile phone penetration are not homogeneous in the country. In order to validate our approach for inferring home and work locations, we turn to the population density data for Côte d’Ivoire ([34] and geonames.org) on a regional and departmental level (as discussed previously, we work with 11 regions in Côte d’Ivoire). The population size per region, correlates with *c* = 0.9633 and *p* < < 0.01 with the aggregated user distribution we have obtained. In addition, our results show a linear relationship on a loglog scale (See supporting material S1 Fig) similar as reported in [25, 26].

As expected, the regions in the north are sparsely populated and in those sub-prefectures we find smallest number of users. In some of them (18 sub-prefectures), Orange does not have any antennas, and there are no users found to live there.

### Mobility can reveal economic activity and service infrastructure

In order to reveal the economic activities and the presence (or absence) of services on a sub-prefecture level in Côte d’Ivoire we are using proxy indicators extracted from the mobile dataset, such as the *average daily trajectory length* of the users in a given sub-prefecture and their *radius of gyration*, see Materials and Methods.

**Fig 4:**
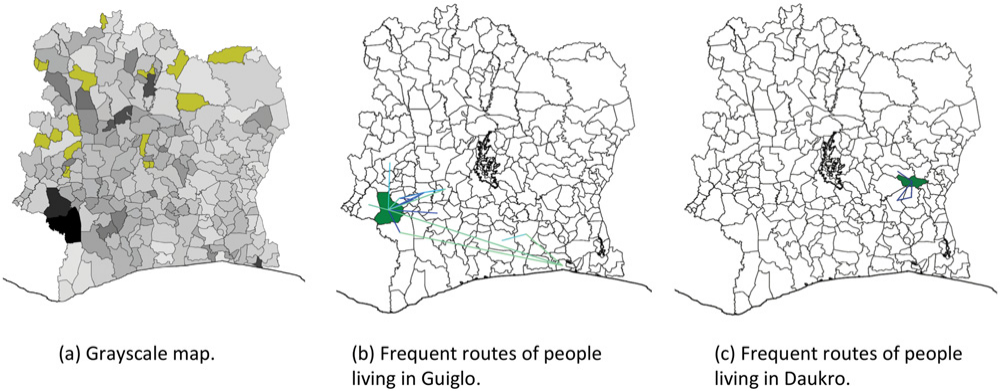
User trajectory statistics. (a) Grayscale map. (b) Guiglo users frequent routes. (c) Daukro users frequent routes.

The map obtained using the average trajectory length (Fig 4(a)), shows that some of the people from the two sub-prefectures in Moyen-Cavally Region tend to travel more on a daily basis (more than 3 times larger distance than the average in the country). The northern of the two sub-prefectures hosts the city of Guiglo which is a market center of the Guere, Yacouba and Mossi people. Guiglo also serves as a depot for timber and coffee that are taken to the ports. Thus, the trajectory length of the people living in those sub-prefectures could be explained by them being traders or truckers. However, we cannot conclude this to be the single reason because a significant amount of the traffic for those people comes from commuting between the two neighboring regions, one of which hosts the national park. In a similar observation, the authors in [35] find unexpected level of activity in the Bas-Sassandra region concerning the incoming and the outgoing call volume per capita. Their results show that the network skirts around this region due to the location of the national park.

In order to gain additional insight into presented observation, we extract the *movement graphs*, see Materials and Methods. In Fig 4(b), we show frequent patterns that emerge in frequent graph mining with threshold 10% from the users who live in Guiglo. In Fig 4(c), we show a similar statistics for the users in Daoukro, a sub-prefecture with a similar number of users found to live there, but a lower average trajectory length. As a conclusion, clear differences in people trajectories in different regions are present, but to understand the reasons behind them, additional information about the people living in these areas would be needed.

**Fig 5:**
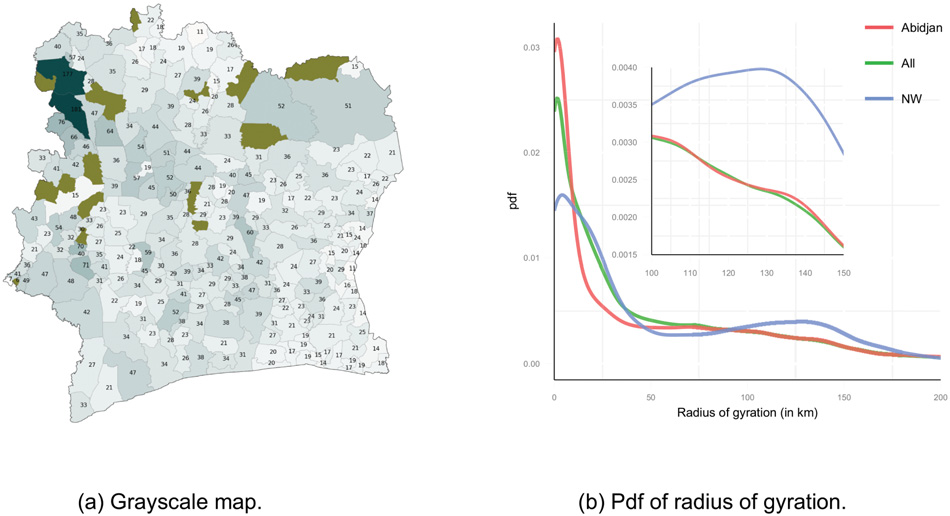
User radius of gyration statistics. (a) Grayscale map. (b) Radius of gyration probability density function.

The gray-scale map in Fig 5(a) presents the average radius of gyration in a given sub-prefecture. This measure shows another interesting pattern of human dynamics in Cote d’Ivoire. Namely, not only that the north-western sub-prefectures of Odienne and Minignan have average radius of user gyration more that 3 times larger than the average in the country, but also the people living in the south-eastern part of the country, in Lagunes and Sud-Comoe regions, have the same radius 2 times lower than the average in the country. The regions in the south-east part of Côte d’Ivoire are those where cocoa, coffee and other agriculture are grown and where most of the industry is concentrated [36]. On the other hand, regions of Savanes, Denguele, Worodougou, Bafing, Moyen Cavaly, part of Zanzan and part of some other regions are less developed. In these regions, even though the economic situation has started improving lately, people sometimes still do not have basic educational and health related services available. The radial increase of the darker color with a center in the south-east of the country clearly shows that the people living in the less developed parts of the country need to travel on a wider radius to the industrial and developed regions in order to fulfill their basic needs, and that the whole country seems to gravitate towards Abijan. At the same time, it is rather interesting how the inhabitants of wealthy southern regions, where also ports and large cities are located, exhibit a smaller average radius of travel, showing lack of need to travel to the northern parts of the country in general (see Fig 5(a)).

The results presented in the map discussed above show that radius of gyration has the property of *spatial-variance*, along with previously reported *time-invariance* [37]. In Fig 5(b) we present further analysis of this interesting property using probability density distribution. The spatial-variance of the probability density distribution can be used as an indicator of the wealth of one region. In Fig 5(b) we have plotted the probability density functions (pdf) of the radius of gyration for the wealthiest region (Abidjan, MPI: 0.17), the poorest region (north east—NE, MPI: 0.53) and the for the whole Côte d’Ivoire. First of all, the radius of gyration for Abidjan in some way dictates the radius of gyration for all the country. However, we see that the travels from the people from the nort east region have much lower probability to be short (i.e. less than 10 km), whereas the probability for longer travels (between 100 and 150 km) is much higher compared to that of Abidjan, see the in-plot in Fig 5(b). This result coincides with the results in [38], where the authors show that the pdf for the agglomerated jump sizes in migration and the radius of gyration in Ivory Coast is more scattered when compared to the one in Portugal. This indicates that the class division is more present on the regional level in Côte d’Ivoire than in, in this case, Portugal.

### Commuting patterns as a measure of poverty

In this part of analysis, we focus on the whole set of users and the whole time period. With our definition of a *commuting trip* (see Materials and Methods), we obtain a graph with 1379 commuting links between 234 (out of 255) sub-prefectures. The data related to socio-economic status is available for 11 development regions in the form of Multidimensional Poverty Index (MPI), that enables to test hypothesis that the poverty can be measured by the number of migration workers from other poles. For each pole, we measure number of workers that commute from other pole during the dataset’s 5 months period. The correlation result with the MPI (*c* = −0.7681, with *p* = 0.0058) shows that our method for extracting commuting patterns can be used to measure the poverty index of a given region. Furthermore, we have obtained better correlation using this method than correlating MPI with the number of calls originating from each region (*c* = −0.7371, with *p* = 0.0096).

### Commuting pattern graph insights

**Fig 6:**
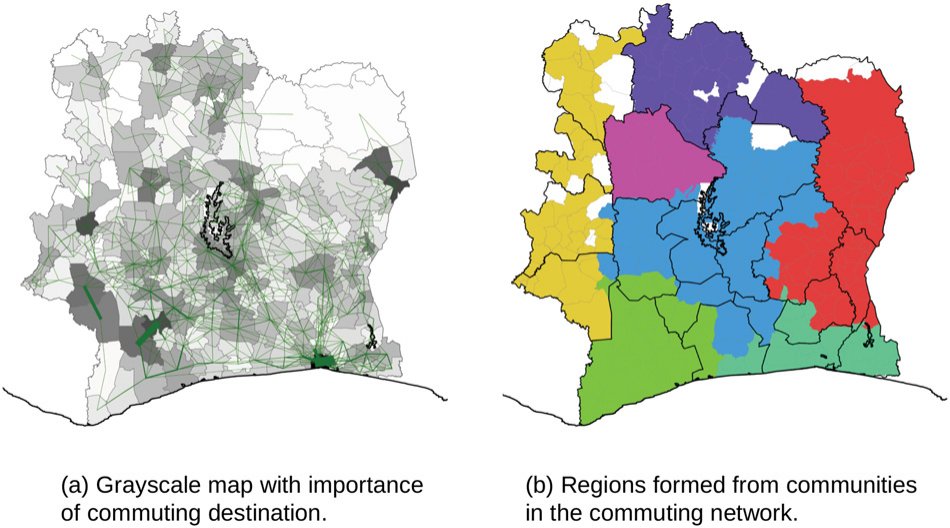
Regions and centers of commuting importance. (a) Grayscale map with commuting centers. (b) Commuting regions map.

Running the PageRank algorithm [39] on the commuting network ranks the sub-prefectures by the importance in the commuting pattern of the country, shown in Fig 6(a). We can see how the cities are ranked higher, as well as pairs of sub-prefectures where intensive commuting happens between them (examples are two sub-prefectures in Moyen-Cavally Region and Soubre and Meaguy in the north of Bas Sassandra that we discussed in previous subsection). Abidjan is obviously the most important commuting center in the country. The thickness of lines between different sub-prefectures scales with the number of observed trips. Precisely, if a trip is taken more times (no matter whether by the same user or different users) this link is shown thicker. These results can provide important initial insight for the traffic planners in the country.

Applying the community detection algorithm [24] on this type of network results in a set of regions of commuting shown in Fig 6(b) (we point out out that the sub-prefectures left in white are those for which we did not find users to have calls in them, so they are left out from the network). The obtained commuting regions follow nicely the borders of administrative regions (or groups of those). For instance, the commuting region shown in yellow corresponds with Denguele region (on the north), and Bafing, Dix-Huit Montagnes, and Moyen-Cavally on the south of it. The pink region almost fully captures Worodougou region, and similarly, the purple commuting region corresponds to the poor Savanes region in the north of the country. The green color region corresponds entirely to Bas Sassandra. However, in the region of Abidjan, the cyan commuting region, the regional borders are blurred and, in addition to Lagunes, we have parts of Sud-Comoe, Sud-Bandama and Agneby captured. This can be explained by the importance of Abidjan as a commuting center for the whole country, particularly for the areas in the vicinity. Similar happens with Yamasuokro, the capital and another important commuting center, capturing parts of Lagunes and N’Zi Comore in the large blue region in the middle. Thus, the overall picture is in agreement with the findings from transportation studies [40], studies using landlines data [41], as well as the other studies using mobile phone data [42, 43], that the administrative regions in a country are a defining factor when it comes to human mobility and interactions. It is important to notice that the authors in [42, 43] discover similar findings using the same dataset as we use, but on a differently defined type of network. On a practical note, our results point to the administrative parties in Côte d’Ivoire for possible need of revising the borders that are distorted by Abidjan and Yamasuokro.

**Fig 7:**
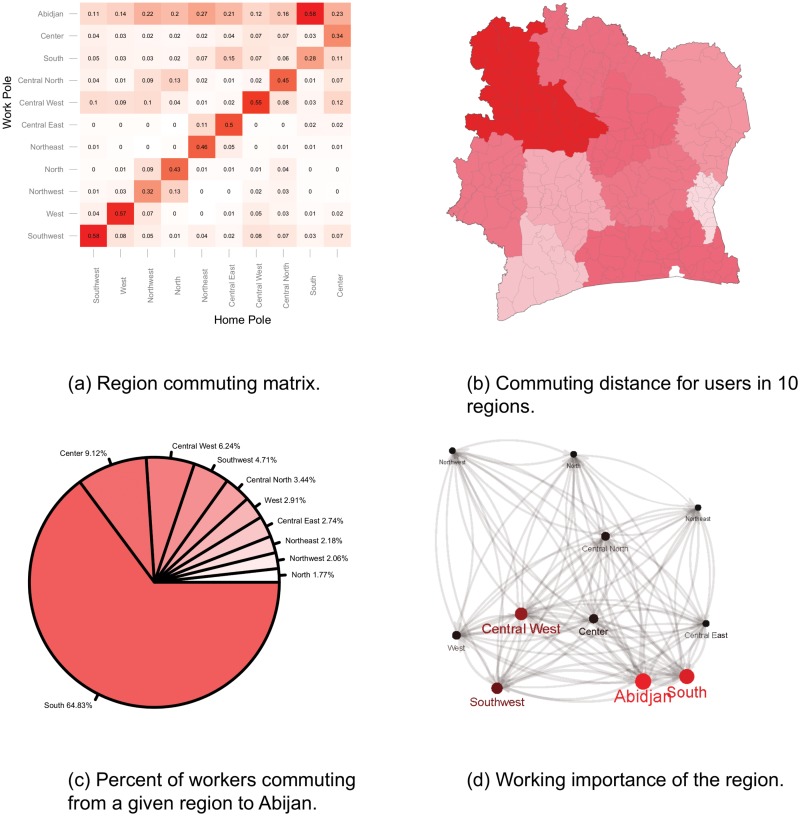
Commuting graph insights. (a) Region commuting matrix. (b) Commuting distance. (c) Percent of commuters to Abijan. (d) Working importance of regions.

In order to give more insights about the commuting ties between the poles we have plotted the commuting matrix in Fig 7(a). The results show strong ‘introvert’ commuting pattern (refer to the main diagonal of the matrix), except for the South region where around 58% of the extracted commuters commute to Abidjan. The most ‘introvert’ commuting, i.e. people tend to work in their home region, was found in the western part of the country, more precisely in the South West, West and the Central West region of Côte d’Ivoire (58%, 57% and 55%, respectively). From the whole number of commuters that work in Abidjan, but live in a different region, around 65% come from the South region, whereas, the smallest fraction is from the Northern regions in the country, mainly due to the big geographical distance (see Fig 7(c)).

Using the matrix from Fig 7(a) and the PageRank algorithm we have plotted the working importance of each region in Fig 7(d). The graph again shows that the most important regions are Abidjan and the South region. In the west part of the country the working center is the Central West, whereas the North part lacks of important working centers.

The commuters from the North East region are the ones that on average travel the most in Côte d’Ivoire, see Fig 7(b). This region is underdeveloped and moreover it lacks of nearby airports, domestic or regional railroad and regional roads. Thus, the investors can either invest in more concentrated gas stations, or build additional railroads or airports with low-cost flights in order to boost the economical development and to have return of investment later.

All of our presented results are obtained solely using the CDR dataset and its comparison to the existing census or other data sources with the hypothesis that the potential exists for using CDR data as a proxy indicator for real world data. We have mostly focused on socio-economic indicators and the high correlation results confirm our hypothesis for a variety of real world data: population density, poverty, urban vs. rural areas, administrative regional borders, power grid and electricity data and important events in the country. Since we analyzed only one dataset from a developing country, our results are not conclusive and ask for similar analyses in countries with different types of economies, size, or region of the world. In addition, we aim to conduct similar comparative studies between the datasets from different countries, as we think they hold potential to reveal more about the universal aspects versus the diversity in human mobility.

## Supporting Information

S1 Fig Population v.s. users found. Per department (left; population data from geonames.org), and per region (right; population data from [34]) on a loglog scale and with fitted linear regression.
(TIFF)
