# Supplementary figures and images for: Mobile Phone Call Data as a Regional Socio-Economic Proxy Indicator
Source: PLoS One. 2015 Apr 21;10(4):e0124160. doi: 10.1371/journal.pone.0124160 (PMC4405276; doi:10.1371/journal.pone.0124160)

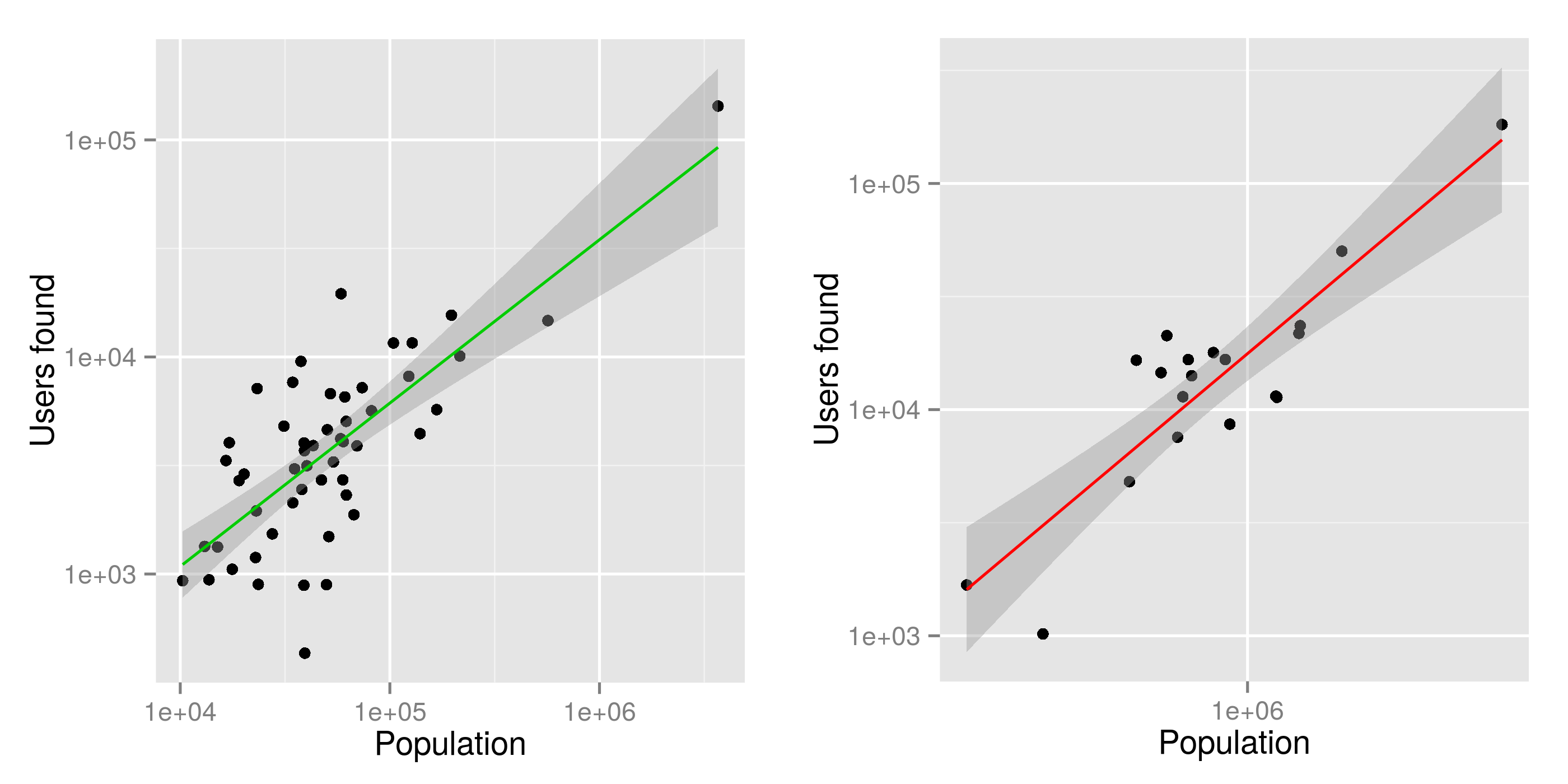

Supplement: S1 Fig — Per department (left; population data from geonames.org), and per region (right; population data from [34]) on a loglog scale and with fitted linear regression. (TIFF) [file pone.0124160.s001.tiff]
